# Kinetics of hepatitis C virus RNA load during pegylated interferon alpha-2a and ribavirin treatment in naïve genotype 1 patients

**DOI:** 10.1186/1476-5926-4-9

**Published:** 2005-12-21

**Authors:** Denis Ouzan, Hacène Khiri, Guillaume Pénaranda, Hélène Joly, Philippe Halfon

**Affiliations:** 1 Arnault Tzanck Institut, Saint Laurent du Var, France; 2 Virological Department, Alphabio Laboratory, Marseille, France; 3 Biostatistics and Epidemiology Department, CDLPharma, Marseille, France

## Abstract

**Background:**

Pegylated interferon given for 24 or 48 weeks constitutes the most effective initial therapy for the treatment of chronic hepatitis C. It has been shown that viral load at week 2 appears the best time for predicting response to treatment. The objectives of this study were to assess whether the hepatitis C virus (HCV) RNA viral decline is predictive of sustained virological response (SVR) and to determine the best time for predicting complete response in our cohort of naïve patients treated with pegylated interferon alpha-2a (Peg-IFN alpha-2a) and ribavirin.

**Results:**

Twenty patients treated with Peg-IFN alpha-2a and ribavirin for 48 weeks were studied. Six months after the end of treatment, a SVR (negative HCV RNA measured by PCR six months after the end of therapy) was obtained in 9 patients. Samples were obtained before and at week 2, 4, 8, and 12. At the end of week 2, viral load decreased more than 1.39 log in 8 out of the 9 patients with SVR and in 1 out of the 11 other patients. When we considered the viral load reduction from baseline to each week of treatment, week 2 appeared to be the best point time for predicting SVR, with a sensitivity of 91% (95%CI: 59;99), a specificity of 89% (52;98), a positive predictive value of 91% (59;99) and a negative predictive value of 89% (57;98).

**Conclusion:**

During treatment with Peg-IFN alpha-2a plus ribavirin in genotype 1 patients, when the main objective of the treatment is viral eradication, viral kinetics showed that week 2 appeared to be the best time point for predicting SVR. Our results must be further confirmed on a larger cohort.

## Background

Interferon alpha plus ribavirin and more recently pegylated interferon (Peg-IFN) given for 24 or 48 weeks constitutes the most effective initial therapy for the treatment of chronic hepatitis C [1-4]. In relapsers, we have previously shown that viral load decline at week 2 appears the best time for predicting the response to treatment [5].

Understanding the kinetics and dynamics of human immunodeficiency virus (HIV) and of hepatitis B virus (HBV) has greatly improved the understanding of the life cycle of these viruses and their response to therapy [6]. Studies of the kinetics of hepatitis C virus (HCV) after initiation of IFN monotherapy have revealed that IFN alpha-2b causes a rapid dose-dependent reduction in HCV RNA levels within 24 to 48 hours. Mathematical calculations revealed that HCV has a serum half-life of 3 hours and a viral production rate of 1.0 × 10^12 ^virions/day [2,7]. This rapid decline appears to be a strong predictor of response to treatment [8,9]. After this rapid decline, there is a slower phase of viral decline that varies widely among patients and is attributed to the death rate of infected hepatocytes. The rate of decline of the second phase, which is probably mediated by immune clearance of infected hepatocytes, appears as the strongest viral kinetic predictor of early viral clearance. Mathematical modelling of viral dynamics revealed that turnover rates of pre-treatment viral production and clearance were high and that in vivo half-lives were a few hours for free HCV virions and 1–70 days for productively infected cells [10]. Infected cell death rate, which determines the second phase decline, is also predictive of response to treatment [11].

In the present study, viral kinetics during Peg-IFN alpha-2a plus ribavirin treatment were determined at week 2, 4, 8, and 12 of treatment in 20 patients after a first course of 48 weeks of Peg-IFN alpha-2a plus ribavirin. Our objectives were to assess: 1) whether the HCV RNA viral decline is predictive of a sustained virological response (SVR); and 2) the best time for predicting complete response in our cohort of patients.

## Results

Epidemiological data for each patient are shown in Table 1. The mean age was (Mean ± SE) 50 ± 11 years and 70% of patients were men. According to Metavir fibrosis staging, 7 patients were staged F0F1 (absent-minimal fibrosis) and 13 were F2-F4 (significant fibrosis to cirrhosis) with one case of cirrhosis (F4).

**Table 1:** Demographics, virological status, and response to treatment of the 20 genotype 1 patients

**ID**	**Sex**	**Age**	**Genotype 1 subtype**	**Liver Biopsy (Metavir)**	**Baseline ALT (IU/L)**	Baseline Viral Load*(log_**10 **_copies/ml)	**HCV RNA viral load decline (log_**10 **_copies/ml)**				SVR**
								
							**W2**	**W4**	**W8**	**W12**	
1	M	45	B	1	58	6.46	0.46	0.74	1.52	2.22	0
2	M	47	-	1	92	5.86	0.92	2.70	3.04	3.07	0
3	F	66	B	3	90	6.56	1.42	3.06	3.06	3.06	1
4	M	53	-	3	57	6.31	1.21	2.69	3.25	2.81	0
5	M	54	B	3	52	5.69	1.66	2.51	2.91	2.91	0
6	M	45	A	2	150	6.88	1.45	3.38	3.38	3.38	1
7	M	55	A	2	92	6.30	1.15	2.63	2.80	3.04	1
8	M	54	A	4	327	6.87	3.37	3.37	3.29	3.29	1
9	F	47	B	0	58	5.78	2.04	2.28	2.28	2.28	1
10	M	63	B	3	218	6.52	2.11	3.02	3.02	3.02	1
11	M	74	-	2	57	7.09	0.65	0.81	1.53	2.18	0
12	M	34	A	2	36	6.79	1.88	3.29	3.29	3.29	1
13	F	54	B	2	75	7.22	0.39	0.98	1.32	1.80	0
14	F	55	B	2	58	7.46	1.32	2.23	3.54	3.96	0
15	F	54	B	1	36	6.31	1.38	2.81	2.81	2.81	0
16	M	44	A	2	39	6.25	1.22	2.40	2.75	2.75	0
17	M	42	A	1	74	6.62	3.12	3.12	3.12	3.12	1
18	M	54	-	2	134	6.38	2.18	2.60	2.88	2.88	1
19	M	38	A	1	65	7.17	0.47	0.66	1.16	1.68	0
20	F	25	A	0	70	5.54	-0.08	0.64	2.04	2.04	0

* Initial viral load Versant™ 3.0 HCV. ** Response, 6 months after the end of the treatment (0 = no response; 1 = response).

**Figure 1:**
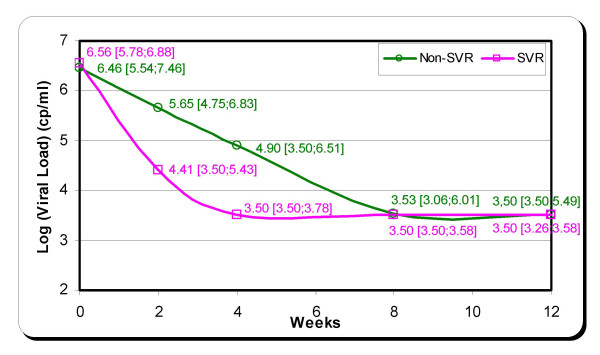
Estimated median [range] viral load decline during the first 12 weeks in patients with (SVR) and without (Non-SVR) sustained response to Peg-IFN alpha-2a + ribavirin. The viral load detection limit of the quantitative assay was 615 UI/ml (3.5 Log).

The difference in logarithmic values of viral load was calculated between the different times: Baseline – Week 2, Baseline – Week 4, Baseline – Week 8, Baseline – Week 12. When we considered the reduction of the viral load (in log_10_) from baseline to each of the first weeks of treatment, week 2 point time tend to be better than other time points for predicting SVR, with an area under receiver operating characteristic (ROC) curve of 0.93 (95% confidence interval: 0.72;0.99), a sensitivity of 91% (59;99), a specificity of 89% (52;98), a positive predictive value (PPV) of 91% (59;99) and a negative predictive value (NPV) of 89% (57;98) (P non-significant) (Table 2). Viral load decline in the patients with SVR and without SVR is shown in Figure 1. Patients with unfavourable virological response had a slight viral load decline of 3 log during the first 8 weeks of treatment. Patients with SVR had the same viral load decline (3 log) in only four weeks of treatment. At the end of week 2, a viral load drop of more than 1.39 log was observed in 8 out of the 9 patients with SVR and in only 1 out of the 11 other patients.

**Table 2:** Diagnostic values and viral drop threshold for Week 2, 4, 8 and 12 analyses.

**Week**	**Viral Drop Threshold (log_**10 **_copies/ml)**	**Area Under ROC Curve (95% CI)**	**Sensitivity (95% CI)**	**Specificity (95% CI)**	**PPV (95% CI)**	**NPV (95% CI)**
2	1.39	0.93 (0.72;0.99)	91 (59;99)	89 (52;98)	91 (59;99)	89 (57;98)
4	2.81	0.89 (0.67;0.98)	100 (71,100)	67 (30;92)	79 (52;92)	100 (61;100)
8	2.81	0.79 (0.55;0.94)	73 (39;94)	78 (40;97)	80 (45;94)	70 (35;85)
12	2.81	0.81 (0.57;0.95)	73 (39;94)	89 (52;98)	89 (52;98)	73 (39:94)

The ROC curve method was used to determine the best cut-off that corresponds to the higher rate of sensitivity and specificity of predictions at week 2, week 4, week 8, and week 12. The accuracy of the test is measured by the area under the ROC curve. This area measures the discrimination, that is, the ability of the test to correctly classify patients with SVR and those without SVR. The analysis shows that the best results are obtained for week 2 viral drop, with a viral drop threshold of 1.39, an area under curve of 0.93, and a sensitivity, a specificity, a PPV, and a NPV rate of respectively 91%, 89%, 91%, and 89%. (P was non-significant for all areas under ROC curves comparisons.)

## Discussion

Viral load measurements provide an indication of viral replication, thereby serving as a valuable tool for guiding the initiation of therapy and subsequent changes. The earliest time to estimate the viral decline that may be predictive of SVR is not clearly defined. Many studies have reported the viral dynamics during the 24–48 hours after treatment initiation [12-17] and have shown that interferon resistance seems to be a dose related phenomenon, rather than an intrinsic characteristic of the virus. Based on pivotal trials in large multicenter studies, positive and negative predictions of SVR using viral load kinetics have been established and now serve as recommendations on antiviral therapy management both by American and by European international consensus conferences. The ability for predicting either a positive or a negative therapeutic response is of an obvious benefit either to clinicians or to patients. Positive predictive evidence early in the course of treatment could be used for reinforcing the importance of compliance in ensuring a successful outcome. Conversely, negative predictive capability would allow clinicians to discontinue therapy early during treatment, which would save health care resources and, even more important, could prevent drug-related adverse events [1-3].

In the present study, when we considered the median reduction of the viral load (in log_10_) from baseline to week 2, 4, 8, and 12 of treatment, week 2 point time tend to be better than other time points for predicting SVR, with a PPV of 91% and a NPV of 89% and a very good sensitivity and specificity (despite a non-significant P-value). Other studies have reported the same observation at week 2 [18-20], whereas Zeuzem *et al*. reported that the absence of a 3 log_10 _viral decline after one month of therapy is associated with an absence of SVR [2]. The low number of patients included in our study can lead to confusing biases on the predictive values; therefore, our results must be confirmed on a larger cohort.

Nine out of the 11 non-responders failed to have SVR after having early virological response (EVR) at week 12 (NPV of 100% and PPV of 50%). These results are in the same range as those of recent studies, which showed that failure to achieve EVR at week 12 of therapy is highly predictive of the absence of therapy (97%–100% negative predictive value), whereas the predictive value of achieving SVR after EVR at week 12 is not so strong (PPV = 65%) [3,21,22].

Methods introduced for analysing HIV dynamics *in vivo *can be modified to give insights into the HCV dynamics, the mechanisms of action of interferon as well as the consequences of varying dosages of interferon [14]. The high turnover rates of HCV explain the rapid generation of viral diversity and the opportunity for viral escape from host immune surveillance and antiviral therapy. Ideally, interferon alpha serum levels should provide constant pressure on the virus and should prevent viral rebound, thereby avoiding continued viral replication and minimising the potential of emergence of drug-resistant quasi-species [23].

## Conclusion

When the main objective of the treatment is viral eradication, the study of viral kinetics during the first twelve weeks of Peg-IFN alpha-2a plus ribavirin treatment showed that week 2 tend to be the best time point for predicting sustained virological response. The early identification of patients with no SVR may lead for proposing an interruption of therapy for avoiding side effects and additional costs, or an early change of therapy [24]. This observation, after being validated on a larger cohort of genotype 1 patients, should have an impact in clinical routine in order to optimise antiviral therapy [25].

## Methods

### Patients

A total of 20 consecutive patients were prospectively enrolled after a first course of combination (Peg-IFN alpha-2a plus ribavirin) and just before starting interferon and ribavirin therapy for chronic HCV infection, at the Hepatology and Gastroenterology Department of the Arnault Tzanck Institute (St Laurent du Var, France). All patients tested positive for HCV RNA. All other causes of chronic hepatitis were excluded by appropriate serological testing and liver histology. All patients were treated with ribavirin 1 g once a week, orally, plus Peg-IFN alfa-2a (180 μg subcutaneous injection once a week) for 48 weeks. Written consent was obtained from each patient, and the study was approved by the local Ethics Committee in accordance with the 1975 declaration of Helsinki. Those patients were all genotype 1. Virological response was assessed by a qualitative HCV RNA assay with a lower sensitivity of 50 IU/ml (HCV Amplicor 2.0 Roche Diagnostics, Meylan, France). According to the qualitative HCV RNA results, patients were defined as virologic sustained responders (HCV RNA negative 6 months after the end of therapy) or as non-responders. SVR was obtained in 9 out of 20 patients. All patients had liver biopsy assessment according to Metavir histological staging.

### Measurement of serum HCV RNA

HCV RNA levels were measured at the same time on blood samples collected at baseline and at weeks 2, 4, 8, and 12 after the initiation of Peg-IFN alpha-2a treatment. Blood was collected in plasma preparation tubes (Becton Dickinson) that were centrifuged directly after collection in order to minimize RNA breakdown [26]. Viral load was assessed using a quantitative bDNA assay (Versant™ 3.0 superscript, Roche, Puteaux, France) with a detection limit of 615 IU/ml (3,200 copies ml). The sensitivity and linearity of the assay were validated for all the genotypes [27]. Genotypes were determined by sequence and phylogenetic analysis of the 5' non-coding of the genome [28]. There were 8 genotype 1A and 8 genotype 1B patients, and, in 4 cases, genotype 1 subtype was undetermined.

### Statistical analysis

The ROC curve method was used to determine the best cut-off between week 2, week 4, week 8, and week 12 viral load measurements, by comparing the areas under the ROC curves. The Hanley-McNeil test was used for testing the statistical significance of the difference between the areas under ROC curves. The significant (alpha) level was set at 5%.

## Competing interests

The author(s) declare that they have no competing interests.

## Authors' contributions

DO was responsible for the conception, the design of the study, and the drafting of the paper. HJ has been involved in drafting and revising the paper HK has been responsible for testing the serums. GP has been responsible for the statistical analysis. PH has been involved in writing the paper. All authors have read and approved the content of this work.
